# Emicizumab utilization, safety, and outcomes in people with severe hemophilia and no inhibitors: 3-year follow-up. A report from the UK Haemophilia Centre Doctors’ Organisation

**DOI:** 10.1016/j.rpth.2025.103164

**Published:** 2025-09-01

**Authors:** Caroline Wall, Hua Xiang, Ben Palmer, Pratima Chowdary, Peter W. Collins, Richard Gorman, Mary Matthias, Charles Percy, Paul Sartain, Susan Shapiro, David Stephensen, Kate Talks, Charles R.M. Hay, UK Haemophilia Centre Doctors’ Organisation (UKHCDO)

**Affiliations:** 1 Department of Haematology, Manchester Royal Infirmary, Manchester, United Kingdom; 2 National Haemophilia Database, Manchester, United Kingdom; 3 Department of Haematology, Royal Free Hospital, London, United Kingdom; 4 Department of Haematology, School of Medicine, Cardiff University, Cardiff, United Kingdom; 5 Brighton and Sussex Medical School, Brighton, United Kingdom; 6 Department of Haematology, Great Ormond Street Hospital, London, United Kingdom; 7 Department of Haematology, Queen Elizabeth Hospital Birmingham, Birmingham, United Kingdom; 8 Patient representative; 9 Department of Haematology, Oxford University Hospitals, United Kingdom; 10 Department of Haematology, Canterbury Christ Church University, Canterbury, United Kingdom; 11 Department of Haematology, Newcastle upon Tyne Hospitals National Health Service Trust, Newcastle upon Tyne, United Kingdom

**Keywords:** efficacy, emicizumab, hemophilia A, non-inhibitor, safety

## Abstract

**Background:**

Emicizumab prophylaxis is restricted to severe hemophilia A in the UK. Treatment choice and safety remain a matter of debate.

**Objectives:**

This study was conducted to investigate factors influencing treatment choice, continued use, safety, and clinical outcomes associated with emicizumab in a national cohort of persons with severe hemophilia A without current inhibitors.

**Methods:**

A 3-year study was conducted in 618 persons with severe hemophilia A who switched to emicizumab and 413 who continued factor (F)VIII prophylaxis. Outcome measures included annualized bleed rates (ABRs), the Hemophilia Joint Health Score, and health-related quality of life.

**Results:**

Switchers and nonswitchers had a similar median age (26 and 28 years, respectively). Switchers had a significantly higher median (IQR) ABR than those continuing FVIII prophylaxis, but a significantly lower proportion had an inhibitor history (13.6% vs 20.5%; *P* = .0005). Thirty-one adverse events were reported, including 1 thrombosis (0.2%), 8/84 recurrent inhibitors (9.5%), 1 neutralizing antidrug antibody (0.2%), and 14/618 (2.3%) patients discontinued emicizumab. A higher prestudy median (IQR) ABR was observed in switchers compared with nonswitchers (2.05 [0.43, 6.06] vs 0.68 [0, 2.7]), reducing to a median (IQR) of 0 (0, 0) with emicizumab prophylaxis. The proportion with a zero-treated bleed rate increased from 35% to 71% (*P* = .001). An 82% reduction in bleeding into target joints was observed in favor of emicizumab. A modest (Δ= −2; *P* = .02) improvement in the total Hemophilia Joint Health Score was observed.

**Conclusions:**

Emicizumab selection was influenced by ABR and inhibitor history but not age. Emicizumab was generally well tolerated, with only 2.3% discontinuing the drug. A significant within-person improvement in all bleeding outcomes was observed with emicizumab.

## Essentials

• We report on a prospective, UK-wide, 3-year postmarketing study of emicizumab prophylaxis.
• Treatment choice was influenced by annualized bleed rate and a past history of a FVIII inhibitor.
• There was a low rate of thrombosis (0.2%) and recurrent inhibitor detection in 8/84 (9.5%) patients.
• Emicizumab reduced the bleed rate and increased the bleed-free proportion from 35% to 71%.

## 1 Introduction

Prophylaxis is the standard of care for people with severe hemophilia A in the UK. The aim is to prevent bleeding, preserve musculoskeletal function, and normalize quality of life [[1], [2], [3], [4]]. Until recently, this involved individualized, regular intravenous infusions of either standard half-life (SHL) or extended half-life (EHL) factor (F)VIII. However, the treatment burden associated with this approach is relatively high, and adherence can be challenging. Although the best results of FVIII prophylaxis are excellent, fewer than 50% of people treated in this way are bleed-free, and progressive arthropathy remains a significant problem [5].

The introduction of emicizumab has changed the hemophilia treatment landscape, with the potential to reduce the treatment burden of prophylaxis through its subcutaneous route of administration and long half-life [6,7]. As a bispecific, humanized, monoclonal immunoglobulin G4 antibody, it mimics FVIII function by bringing FIXa and FX into proximity [8]. The safety and efficacy of emicizumab were reported pre-licensure clinical trial program in people with and without inhibitors [7,[9], [10], [11], [12]]. However, the proportion of trial subjects using FVIII prophylaxis prior to emicizumab was relatively low, children under 12 were underrepresented, and those with a history of treated inhibitors within 5 years or a history of thrombotic events were excluded [9]. Although generally well tolerated, it is not known what proportion of individuals switch back to FVIII prophylaxis [7]. There are persistent concerns about an uncertain potential thrombosis risk, especially in the elderly [13,14].

These real-world postmarketing studies provided safety and efficacy data for a large and diverse cohort in which treatment selection was restricted only by the product license. This offers the opportunity to investigate factors influencing treatment choice and reasons for switching back to FVIII prophylaxis, as well as safety, real-world efficacy, and medium-term clinical outcomes. The National Hemophilia Database (NHD) monitors and reports the usage, efficacy, and safety of all products used to treat bleeding disorders in the UK through a centralized collection of hemophilia center-derived and patient-reported outcome data. This provides an opportunity to evaluate emicizumab outcomes and compare them with those of previous treatments and with outcomes in people with severe hemophilia A continuing to use factor prophylaxis.

## 2 Methods

### 2.1 Study design and data collection

The NHD is a centralized UK database designed to collect data on the diagnosis, management, and complications of people with bleeding disorders in the UK. Baseline diagnostic and demographic details are collated and updated quarterly, including weight, inhibitor status, and the treatment issued by hemophilia centers.

This national postmarketing study was designed to determine the safety and efficacy of emicizumab prophylaxis in people with severe hemophilia A without reported current inhibitors in normal clinical practice between January 1, 2019, and December 31, 2022. Emicizumab was prescribed within the licensed indication but otherwise at the discretion of the local hemophilia center. Dosage was monitored by hospital pharmacies, and a guidance document was issued prior to the commissioning of emicizumab to provide guidance on optimal combinations of vial sizes to minimize wastage and overprescribing. The duration of overlap of FVIII concentrate during the emicizumab loading period varied from 0 to 3 weeks and was at the discretion of the managing clinician. Most had no overlap period. Children under 3 years at the time of their first treatment will be reported separately.

**Figure 1:**
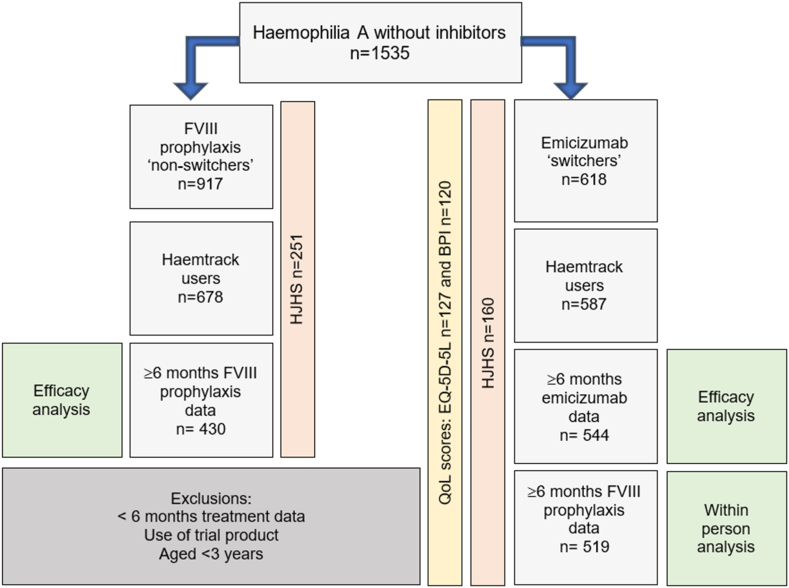
CONSORT diagram illustrating disposition of subjects. Efficacy analysis included. BPI, Brief Pain Inventory; CONSORT, consolidated standards of reporting trials; EQ-5D-5L, EuroQoL 5-Dimensions 5-Levels questionnaire; FVIII, factor VIII; HJHS, Hemophilia Joint Health Score; QoL, quality of life.

People with severe hemophilia A who were first issued with hemophilia treatments prior to 2021 were assessed, and those who had switched to emicizumab (“switchers”) and had at least 6 months of compliance reported treatment issue data before and after the quarter in which they switched by the study endpoint (2022 quarter 1), were identified. People with severe hemophilia A who had not switched to emicizumab by the study endpoint (“nonswitchers”) were assigned a “dummy” switch quarter (2020 quarter 4) based on the midpoint of the introduction of emicizumab for people with severe hemophilia A without inhibitors in the 2019 quarter 3 and the study endpoint of the 2022 quarter 1. Nonswitchers with at least 6 months of treatment issue data before and after the “dummy” switch quarter were identified. People with severe hemophilia A with inhibitors at the time of switch, aged under 3 years at switch, issued trial products within 2 years preswitch or 1 year postswitch, or previously described in Wall et al. [15], were excluded (Figure 1).

A smartphone application home therapy diary, Haemtrack (HT), was used to record treatments and bleeding data (including chronological details, treatment indication, product type, and dose used). HT only records treated bleeds, and untreated bleeds are not recorded. These systems and data validation steps have previously been described [16]. This minimized the effect of potential confounders.

A subanalysis of bleeding outcomes required at least 6 months of self-reported HT data, both before and after switching for switchers, and at least 6 months of HT data for nonswitchers between September 1, 2019, and March 31, 2022.

The cohort that continued FVIII prophylaxis (“nonswitchers”) is described, and bleeding outcomes are reported for those who were compliant with treatment. Definitions of treatment compliance in this context have been described by Hay et al. [16]. Within-person comparison of bleed outcomes with emicizumab and prior FVIII prophylaxis was conducted. The comparison between the emicizumab-treated cohort and the cohort continuing FVIII prophylaxis was performed only to investigate factors influencing treatment choice, since the 2 groups were neither matched nor selected randomly.

Adverse events were reported electronically to the NHD and were investigated and evaluated by the Adverse Events Panel of the UK Haemophilia Centre Doctors’ Organisation. Current inhibitor status was reported quarterly by the local hemophilia center to the NHD, and only people reported as having no current inhibitor at study entry were included. The detection of a new or recurrent inhibitor after study entry was recorded as an adverse event. The presence of antidrug antibodies was inferred from falling drug levels and/or emergence of breakthrough bleeding and confirmed by a centrally conducted antibody assay at the University College Hospital using GloBody technology [17]. All adverse events were reported monthly to the manufacturer and regulators. Discontinuation of emicizumab was similarly investigated.

### 2.2 Objectives and outcome measures

The primary outcome measure of efficacy was the treated annualized bleed rate (ABR). Secondary endpoints included ABRs, annualized spontaneous bleed rates, the proportion of people with zero-treated bleeds, the presence of target joints, change in the total Hemophilia Joint Health Score (HJHS), and health-related quality of life measures. Target joints were defined by the International Society on Thrombosis and Haemostasis criteria and derived from HT data [18]. The primary safety objectives were to evaluate the frequency of thromboses, including thrombotic microangiopathies. Surveillance for FVIII inhibitors was performed according to clinical guidelines and using bovine chromogenic reagents [4,19]. New or relapsed inhibitors were reported to the NHD. Emicizumab drug levels were monitored routinely. Neutralizing antidrug antibodies were tested if suspected on clinical or laboratory grounds. Secondary safety outcomes included other adverse events.

Joint health was assessed using the HJHS version 2.1, and data were collected centrally [20]. Participants with an HJHS within the 2 years prior to starting emicizumab (T0) and a paired score at least 6 months (T1) after starting emicizumab were included. A change in the total HJHS of ≥4 was considered clinically meaningful [21]. HJHS with 4 or more missing items at either data point were excluded [18].

The impact of emicizumab on health-related quality of life was evaluated using EuroQol 5-Dimensions 5-Levels (EQ-5D-5L) and Brief Pain Inventory (BPI) questionnaires completed in paper format at baseline and 6-monthly thereafter [22,23]. The EQ-5D-5L is composed of 5 self-rated qualitative dimensions and a quantitative visual analog scale of overall health. The BPI provides records of pain intensity and interference with function.

### 2.3 Statistical analysis and study conduct

Descriptive statistics (medians, IQRs, and arithmetic ranges) are used to summarize baseline demographics and bleeding outcomes. Within-person comparison of the ABRs, annualized joint bleed rates, and HJHS was performed using the Wilcoxon matched-pairs signed-rank test, and zero-treated bleed rates were calculated using the chi-square McNemar test. Zero-treated bleed rates are reported over matched timeframes (52 weeks) to avoid misinterpreting favorable outcomes with shorter follow-up. Stata statistical software release 11 (StataCorp), RStudio (Posit), and Prism 9 (GraphPad) were used to perform the analyses. Observational research conducted on the database was permitted by ethical approval granted by the National Health Service Health Research Authority North-West – Haydock Research Ethics Committee (Research Ethics Committee reference 19/NW/0009, Integrated Reasearch Application System project ID: 252831).

## 3 Results

### 3.1 Study population and treatment choice

Following exclusion criteria, 1535 people with severe hemophilia A without an inhibitor were included in the study, of whom 618 (40.3%) switched to emicizumab prophylaxis and 917 (59.7%) continued treatment with prophylactic or on-demand FVIII (Figure 1, Table 1).

**Table 1:** Demographics of UK individuals with severe hemophilia A without a current inhibitor-issued product during the study period.

Subject characteristics	Nonswitchers *n* = 917	Switchers *n* = 618
Age (y), median (IQR) [range]	28 (17, 40) [3, 82]	26 (12, 41) [3, 79]
3-17, *n* (%)	238 (26.0)	217 (35.1)
18-29, *n* (%)	257 (28.0)	126 (20.4)
30-49, *n* (%)	281 (30.6)	179 (29.0)
≥50, *n* (%)	141 (15.4)	96 (15.5)
History of an inhibitor, *n* (%)		534 (86.4)
No	729 (79.5)	84 (13.6)
Yes	188 (20.5)	*P* = .0005
Preswitch ABR, median (IQR)		
Age < 18 y	0.0 (0.0, 1.4) *n* = 148	1.3 (0.3, 3.0) *n* = 176
Age ≥ 18 y	0.7 (0.0,3.3) *n* = 282	2.8 (0.6, 8.5) *n* = 343

People with insufficient Haemtrack data to analyze bleeding outcomes were excluded from the ABR results.

ABR, annualized bleed rate.

Treatment choice was restricted only by the product license and appeared to be influenced by current ABR and past FVIII inhibitor history, but not by age. In total, a past inhibitor history was reported in 272 individuals, of whom 84 (31.1%) switched to emicizumab, while 188 (68.9%) continued FVIII replacement (*P* = .0005). Emicizumab was prescribed to 40% of potentially eligible people with severe hemophilia A: age 3 to 17 years: 48%; 18 to 29 years: 33%; 30 to 49 years: 39%; and >50 years: 40.5%.

### 3.2 Adverse events, drug discontinuation, and recurrence of inhibitors

In total, 31 adverse events were reported in 28/618 (4.5%) emicizumab-treated individuals (Table 2). Drug-related adverse events occurred predominantly in the first 6 weeks of treatment. Adverse events included 5 deaths, causally unrelated to treatment. There were no reports of thrombotic microangiopathies or coprescription of factor eight inhibitor bypassing activity. Events of special interest are summarized in Table 2.

**Table 2:** Adverse events and drug cessation reported to the National Hemophilia Database in people with severe hemophilia A without inhibitors prescribed at least 1 dose of emicizumab (denominator *n* = 618).

Adverse event	Frequency *n* (%)	Drug discontinuation *n* (%)
Cutaneous reactions	5 (0.8)	3 (0.48)
Headache	5 (0.8)	4 (0.65)
Arthralgia	2 (0.32)	2 (0.32)
Thrombosis	1 (0.16)	1 (0.16)
Intracranial hemorrhage	1 (0.16)	0 (0.16)
Poor efficacy	1 (0.16)	1 (0.16)
ADA	1 (0.16)	1 (0.16)
FVIII inhibitor recurrence^a^	8 (9.4)	0
Death	5 (0.8)	-
Other	1 (0.2)	0
Patient choice	-	1 (0.2)
Poor compliance	1 (0.2)	1 (0.2)
Total	**31**	**14**

ADA, antidrug antibody; FVIII, factor VIII.

a Inhibitor recurrence denominator *n* = 84.

### 3.3 Drug discontinuation

A total of 14 (2.3%) people who had at least 1 dose of emicizumab switched back to FVIII prophylaxis during the study period (Table 3). Reasons given for discontinuing emicizumab included moderately severe systemic drug reactions (*n* = 3) and postinfusion headache (*n* = 4). These side effects occurred in the first 6 to 8 weeks of use and were considered drug-related. Two patients discontinued emicizumab because of the emergence of seronegative arthralgia of small joints, which did not resolve after cessation of emicizumab, and therefore, their relationship with treatment is uncertain. Other reasons for discontinuation of emicizumab are listed in Table 2.

**Table 3:** Within-person comparison of bleeding outcomes for switchers and efficacy analysis of nonswitchers by factor VIII product type.

Subject characteristics	Switchers				Nonswitchers	
	SHL *n* = 406		EHL *n* = 113		SHL *n* = 301	EHL *n* = 129
	Preswitch	Postswitch	Preswitch	Postswitch		
Age (y), median (IQR)	29 (14, 43)		20 (11, 40)		27 (15, 39)	22 (11, 40)
3-17, *n* (%)	124 (31)		52 (46)		100 (33)	48 (37)
18-29, *n* (%)	80 (20)		25 (22)		67 (22)	31 (24)
30-49, *n* (%)	132 (33)		20 (18)		88 (29)	26 (20)
≥50, *n* (%)	70 (17)		16 (14)		46 (15)	24 (19)
Follow-up (wk), median (IQR)	167 (151, 199)	85 (61, 110)	166 (148, 199)	86 (70, 114)	77 (72, 77)	77 (77, 77)
Infusion frequency/wk, median (IQR)	2.73 (1.57, 3.49)		2.27 (1.86, 2.76)		3.22 (2.52, 3.55)	2.09 (1.88, 2.53)
Dose (U/kg/wk), median (IQR)	64 (42, 93)		66 (52, 93)		84.3 (56, 117)	78.8 (56, 107)
ABR, median (IQR)	2.32 (0.62, 6.81)	0.00 (0, 0.7)	1.40 (0.28, 4.66)	0.00 (0.00, 0.72)	0.68 (0.00, 2.90)	0.68 (0, 2.67)
ASBR, median (IQR)	0.77 (0.00, 3.05)	0.00 (0, 0)	0.32 (0, 1.41)	0.00 (0.00, 0.00)	0.00 (0.00, 1.33)	0.00 (0.00, 0.68)
AJBR, median (IQR)	1.37 (0.32, 4.15)	0 (0, 0.46)	0.61 (0, 1.87)	0.00 (0.00, 0.00)	0.00 (0.00, 1.51)	0.00 (0.00, 1.35)
Zero-treated bleeds,^a^*n* (%)	131 (32)	290 (71)	52 (46)	79 (70)	155 (51)	62 (48)

Bleeding outcomes with emicizumab prophylaxis are compared with previous factor VIII prophylaxis in people with >6 months preswitch and postswitch Haemtrack data. Infusion frequency and dosage figures are derived from reports of the hemophilia centers.

ABR, annualized bleed rate; AJBR, annualized joint bleed rate; ASBR, annualized spontaneous bleed rate; EHL, extended half-life prophylaxis; SHL, standard half-life prophylaxis.

a Zero-treated bleed rates are matched over 52 weeks.

### 3.4 Recurrence of FVIII inhibitors

Detectable inhibitors were reported after changing to emicizumab in 8/84 (9.5%) individuals who had a past history of an inhibitor and a negative Bethesda assay when they started emicizumab. None of these patients was treated with FVIII between starting emicizumab and the detection of the inhibitor after switching. In 5/8 (5.9%) cases, pharmacokinetic and clinical data indicated the probable presence of a low-level inhibitor despite a negative Bethesda assay, when patients switched to emicizumab, suggesting incomplete tolerance. A measurable inhibitor was reported in this group at 2, 3, 4, 12, and 16 months after switching to emicizumab, without loss of efficacy of emicizumab. Peak inhibitor titers in this group were 0.4, 1.3, 1.3, 1.7, and 44 Bethesda Units/mL, respectively.

Inhibitors recurred in 3 (3.6%) people who were considered to have been successfully tolerized for 4, 10, and 15 years before switching, respectively. These were first detected at 2, 2, and 4 months after switching to emicizumab with a peak inhibitor titer of 1.3, 1.6, and 1.7 Bethesda Units/mL, respectively.

### 3.5 Antidrug antibody and poor efficacy

One case of antidrug antibody was reported and detected during routine surveillance, suggested by a reduction in emicizumab levels (14-20 mcg/mL) with a normalized activated partial thromboplastin time, but without associated loss of efficacy. Factor prophylaxis was restarted at the individual’s request. There were 2 reports of poor efficacy without evidence of an antidrug antibody. One patient resolved with improved drug compliance, and the second patient reverted to prophylaxis with EHL FVIII.

### 3.6 Thrombosis

Bilateral renal infarcts were diagnosed in a 54-year-old male, with fibromuscular dysplasia of the renal arteries, who developed hematuria 3 days after the third loading dose of emicizumab. He had no other thrombotic risk factors. Although this was primarily attributed to fibromuscular dysplasia of the renal arteries, emicizumab was thought likely to be a contributory factor and was stopped. FVIII had been stopped more than a week before the onset of hematuria.

### 3.7 Intracranial hemorrhage

A nontraumatic hemorrhagic stroke in the deep pons, which resulted in death 14 days after presentation, occurred in a 51-year-old male presenting with hemiplegia. The event was attributed to severe hypertension. He had been bleed-free for 2 years since starting emicizumab, and emicizumab levels on admission were 80 mcg/mL. The event was considered unrelated to emicizumab.

### 3.8 Efficacy of emicizumab

Compliant HT data were available for 544 (88%) emicizumab-treated individuals, of whom 519 (84%) had at least 6 months of FVIII prophylaxis data immediately preswitch (Table 3).

Before changing to emicizumab, 406 (78.2%) patients used SHL and 113 (21.8%) used EHL FVIII prophylaxis. Preswitch FVIII dosage and infusion frequency are shown in Table 3. The preswitch median (IQR) ABR was 2.1 (0.4, 6.1) with FVIII prophylaxis, and 35% of patients reported zero-treated bleeds in the 52 weeks before switching to emicizumab. After changing to emicizumab, the median (IQR) ABR reduced to 0.0 (0.0, 0.7), and 71% of patients reported no bleeds (*P* < .001). Emicizumab resulted in an overall 49.6% increase in the proportion of patients reporting zero-treated bleed rates; 80% of children and 67% of adults were “bleed-free.” Breakthrough bleeding occurred in 150 people (36%); the median (IQR) age was 36 (15, 50) years. The change to emicizumab in this subgroup was associated with a 72.9% reduction in ABR, from a median (IQR) of 4.2 (1.7, 9.8) events to 1.1 (0.3, 2.2; *P* < .01).

### 3.9 FVIII prophylaxis cohort (“nonswitchers”)

In total, there were 917 nonswitchers, of whom 430 (46.9%) had compliant HT FVIII prophylaxis data (Table 3). SHL products were used in 301 (70.0%) people, and EHL in 129 (30.0%) people with severe hemophilia A (Table 3). The median (IQR) ABR in children was 0.00 (0.00, 1.35), and 59% reported zero-treated bleeds. In adults, this was 0.68 (0.0, 0.32) and 46%, respectively. The median ABR was 0.68 for both SHL and EHL prophylaxis, but patients on EHL prophylaxis had 1.13 fewer infusions per week than those using SHL FVIII.

### 3.10 Target joints

**Figure 2:**
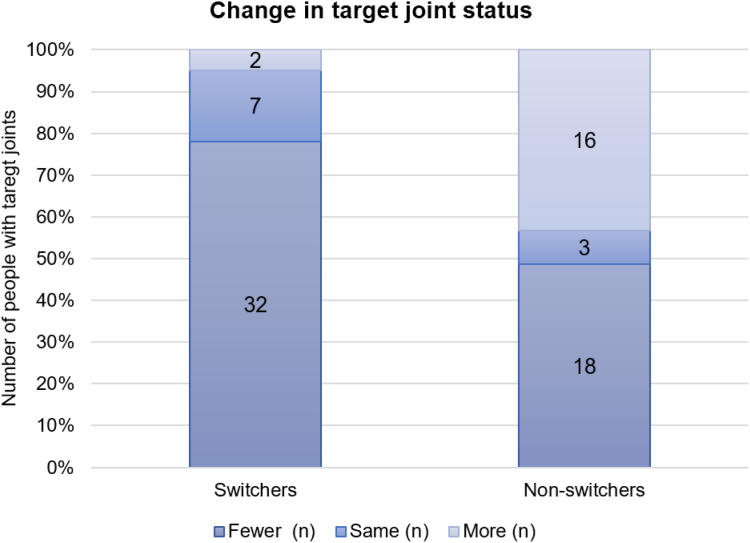
Change in target joint status. Proportion of individuals recording fewer, the same, or more target joints over 90 weeks (absolute values are labeled).

The median (IQR) age of people with severe hemophilia A and the number of target joints at baseline were similar in both switchers and nonswitchers, at 41 (31, 47) and 41 (33, 51) years, respectively. Among those transitioning to emicizumab, 38 individuals (7.3%) had 56 target joints at baseline, and after a median follow-up of 85 weeks, 9 individuals (1.7%) exhibited 10 target joints, and 46/56 (82.1%) of the target joints had resolved. In individuals continuing FVIII prophylaxis, 21 people (5.0%) had 25 target joints at baseline, and after a median follow-up of 77 weeks, 18 individuals (3.1%) showed 23 target joints, ie, only 2/25 (8%) had resolved. A higher proportion of emicizumab switchers experienced a reduction in the number of target joints over time compared with people continuing FVIII prophylaxis (Figure 2). Comparing the target joint rates between switchers and nonswitchers at baseline, the *P* value was .16. Postswitch, the *P* value was .04.

### 3.11 HJHS

**Figure 3:**
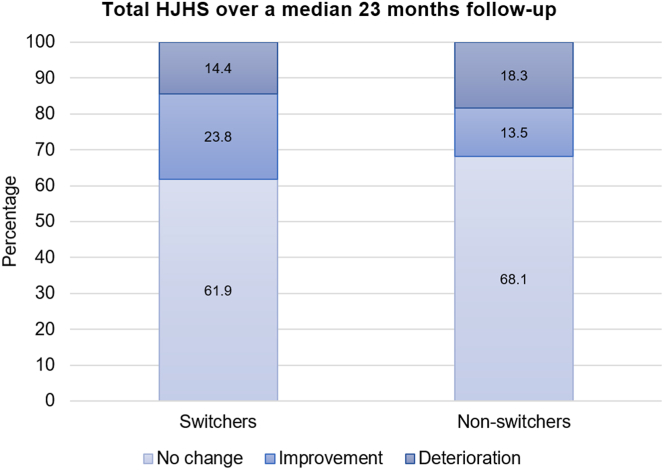
Hemophilia Joint Health Scores (HJHS) over a median of 23 months of follow-up. A difference in total HJHS between baseline and end of study of under 4 was categorized as "no change," a reduction of 4 or more as "improvement," and an increase of 4 or more as "deterioration."

Paired HJHS data were available for 160 (25.9%) and 251 (27.4%) of switchers and nonswitchers, respectively, with a median interval between T0 (baseline) and T1 (latest) scores of 23.1 months. The cohorts in this subset were not formally matched, but they had very similar median (IQR) ages at the study outset (28 [14, 45] and 27 [15, 40] years, respectively). Increased age was associated with a higher T0 total HJHS. Children aged <18 years had a median (IQR) total HJHS of 0 (0, 2), which increased to 27 (14, 41) in those aged >30 years (*P* = .41). In emicizumab switchers, the median (IQR) T0 total HJHS was 9.5 (1.0, 28.5) and 7.5 (0.0, 26.8) at follow-up (*P* = .02), respectively, and in nonswitchers, it was 7 (0, 27) and 7 (0, 27), respectively. An increase or decrease in total HJHS > 4 (Figure 3) or individual joint level HJHS > 2 is considered clinically relevant. There was a significant difference in the proportion of switchers with improved left elbow (17/158, 10.8%) compared with nonswitchers (9/251; 3.6%; *P* = .005). There appeared to be a relative, nonsignificant improvement in other joints for switchers compared with nonswitchers.

### 3.12 Quality of life

#### 3.12.1 EQ5D 5L

**Figure 4:**
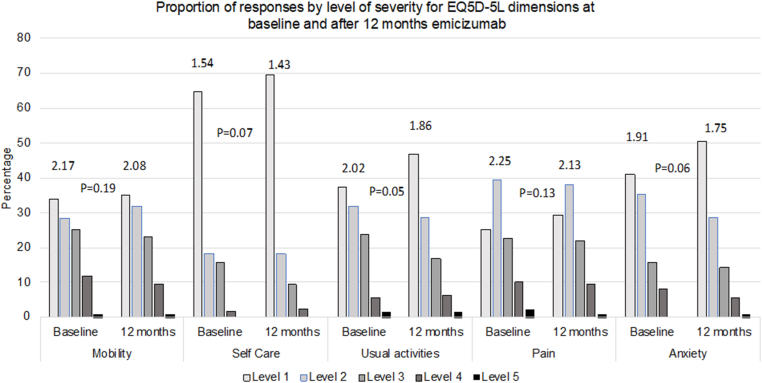
Proportion of responses by level of severity for EuroQoL 5-Dimensions 5-Levels (EQ5D-5L) at baseline and after 12 months of emicizumab prophylaxis. Mean domain scores are displayed. Level 1 = no problems, level 2 = slight problems, level 3 = moderate problems, level 4 = severe problems, and level 5 = extreme problems/unable.

Paired scores at baseline and after 12 months of emicizumab prophylaxis were available for 127 individuals. Problems with pain, mobility, and usual activity domains were most frequently experienced. A summary of the proportion of responses by level of severity at baseline and after 12 months of emicizumab is shown in Figure 4. There were nonsignificant trends toward improvement in all domains. At baseline, 16 (12.6%) individuals reported no problems in any domain (health profile 11111), which increased to 24 (18.9%) at 12 months. The median (IQR) visual analog scale score increased from 75 (65, 90) at baseline to 80 (65, 90) at 12 months, *P* = .3.

#### 3.12.2 BPI

**Figure 5:**
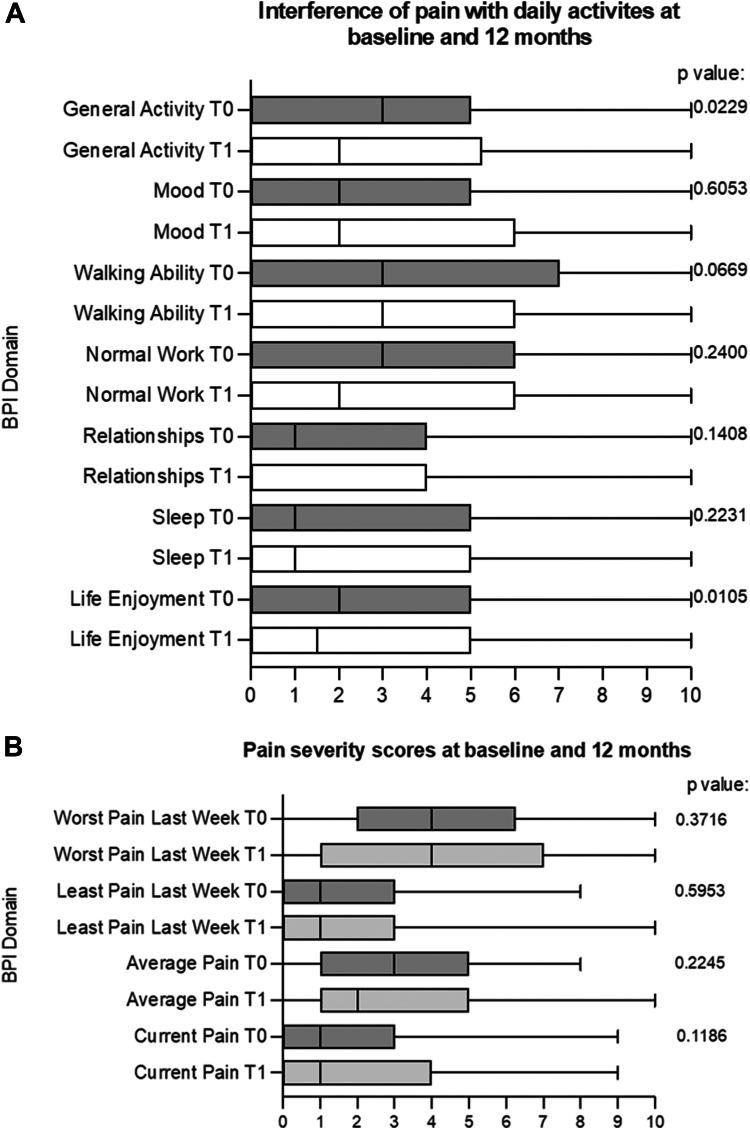
Brief Pain Inventory (BPI) score box and whisker plots. Baseline scores are labeled T0 and 12-month scores T1. Medians, IQRs, and ranges are plotted. Comparison of T0 and T1 scores was by the Wilcoxon matched-pairs signed-rank test. (A) Pain severity as reported over the previous week on a 0 to 10 scale (0 = no pain, 10 = worst pain). (B) The impact of pain on 7 aspects of daily life on a scale of 0 to 10 (0 = not at all, 10 = extreme impact).

In total, 120 emicizumab-treated individuals completed paired BPI questionnaires. The “average pain” score at baseline was a median (IQR) of 3 (5, 1) and decreased to 2 (5, 1) after 12 months of treatment (*P* = .22). Pain interfered most with walking ability, normal working, and general activities (Figure 5). Improvements in general activity (*P* = .02) and life enjoyment (*P* = .01) scores were statistically significant.

## 4 Discussion

In this UK postmarketing study of emicizumab prophylaxis, treatment choice was not restricted by cost or treatment guideline but was prescribed at the managing clinician’s discretion and patient choice. The uptake of emicizumab varied considerably between hemophilia centers, reflecting a lack of consensus around treatment selection [24].

While qualitative information on treatment choice was not collected, our data suggest that therapeutic choice was influenced by the bleed rate and FVIII inhibitor history of people with severe hemophilia A, but not by age. Higher baseline bleeding rates and greater prevalence of target joints were observed in switchers, suggesting that those considered to be doing well with FVIII prophylaxis were less likely to change to emicizumab, despite the lower treatment burden associated with emicizumab. People continuing conventional prophylaxis generally used more intensive FVIII replacement than those who chose emicizumab (median, 82.6 U/kg/wk compared with 64 U/kg/wk).

Increasing age did not appear to influence treatment choice; 40% of people with severe hemophilia A aged >50 were prescribed emicizumab.

A higher proportion of those continuing FVIII prophylaxis had a history of inhibitors (20.5% vs 13.6%; odds ratio, 0.61; 95% CI, 0.46-0.8; *P* = .005), suggesting that the risk of inhibitor recurrence may be a factor influencing the choice of emicizumab.

Laboratory surveillance revealed a relatively high incidence of inhibitor recurrence in 8/84 (9.5%) people with a past history of an inhibitor. There was evidence of a persistent low-level inhibitor, despite the negative Bethesda assay, prior to starting emicizumab in 5 of these individuals, based on reduced FVIII half-life. In a further 3 cases, inhibitors were detected 2 to 4 months after starting emicizumab and 4 to 15 years postimmune tolerance induction (ITI). This event of special interest was not addressed in the HAVEN studies, which excluded subjects with an inhibitor detected in the previous 5 years [25]. Isolated cases of inhibitor recrudescence after starting emicizumab have been reported, predominantly in partially tolerized individuals. Inhibitors are known to relapse many years after apparent ITI [[26], [27], [28], [29], [30]]. It has been postulated that regular FVIII exposure may be needed to maintain tolerance after ITI, although definitive evidence for this is lacking, and relapses may occur despite ongoing FVIII exposure [[31], [32], [33]]. Since none of these cases were treated with FVIII between changing to emicizumab prophylaxis and inhibitor detection, it would appear that withdrawal of FVIII prophylaxis was the key event leading to an increase in inhibitor titer. Allowing for the limitations of small numbers, this study shows that the risk of inhibitor recurrence was about 4% in people with severe hemophilia A who had a normal FVIII half-life and 9.5% in those with evidence of low-level inhibitor activity and a reduced FVIII half-life. Fortunately, recurrent inhibitors were, with one exception, of low titer. The risk of inhibitor recurrence should be discussed prior to changing products. The incidence and context of this complication are an important element of that conversation. The immunogenicity of emicizumab itself is relatively low. Only 1 fully neutralizing antidrug antibody was reported in this series, though partly neutralizing antibodies, which do not affect drug efficacy, were undoubtedly common, findings that are consistent with the HAVEN study [34].

We observed a significant within-person improvement in bleeding outcomes relative to previous FVIII prophylaxis. Spontaneous bleeding was infrequent, and the proportion reporting zero-treated bleeds increased from 35% to 71% (*P* < .01). The subset of 29% of people with severe hemophilia A who reported breakthrough bleeding during emicizumab prophylaxis experienced a 73% reduction in ABR compared with prior treatment. This subset was generally older with higher baseline bleeding rates.

The prevalence of target joints at study entry was low relative to clinical trials (6.5% vs 67%) because prophylaxis has long been the UK standard of care [9]. The use of emicizumab prophylaxis was associated with an 82% reduction in bleeding into target joints, supporting clinical study findings [35]. Joint health was generally well preserved in children, but progressively higher total HJHS occurred with age. A modest (δ= −2; *P* = .02) reduction in total HJHS was observed over 2 years of emicizumab prophylaxis; however, the reduction was less than 4, which has been reported to be clinically relevant [21]. It is not known if the HJHS will improve to a meaningful level with ongoing emicizumab prophylaxis beyond 2 years. Further follow-up is required to determine the long-term effect of emicizumab on joint health. Reporting of the HJHS was incomplete and was impacted by the COVID-19 pandemic. Furthermore, the HJHS was developed for use in children, and joint scoring is relatively insensitive to subtle changes, which may result in a ceiling effect, particularly in people with preexisting arthropathy. Although the HJHS in our data could have been influenced by between-physiotherapist variability, we have attempted to minimize this variability by regular HJHS training of our physiotherapists.

Reducing the treatment burden of prophylaxis was anticipated to improve quality of life. While statistically significant improvements in the BPI domains of “interference of pain with general activities” and “life enjoyment” were observed, changes in EQ-5D-5L were not significant. Phase 3 trials of people with severe hemophilia A without inhibitors demonstrated improvements in physical health scores, but only in people with the poorest preswitch bleeding control [6]. The patient-reported outcomes selected were chosen for their ease of use and widespread use, but they appear insensitive to changes in treatment burden.

This is the largest real-world study of emicizumab prophylaxis to date. Observation over a robust duration of time in a large cohort improved the reliability of outcomes and the detection of infrequent events. Despite this, follow-up was less structured in comparison with clinical trials, and not all data sets were complete. However, the methodological limitations common to observational studies were mitigated where feasible. Access to emicizumab was conditional on HT use, which promoted high rates of engagement. Furthermore, confounding biases that may affect parallel group comparisons were minimized by the use of within-person analysis, which minimized the effect of variation in reporting bleeds. It is suspected that mild, self-limiting adverse events, such as localized reactions, were underreported to the NHD, but significant events were more completely described.

This study provides additional insight into the comparative efficacy of emicizumab with real-world factor prophylaxis. Emicizumab was associated with a significant reduction in bleeding events, in comparison with prior treatment, in a large cohort who may have been switched partly due to suboptimal bleeding outcomes with factor prophylaxis. The incidence of thrombosis in this cohort was very low despite widespread use in older age. This is the first report to quantify the incidence of inhibitor recurrence after cessation of regular factor exposure, which may inform discussions with people considering starting emicizumab.

## References

[1] Nilsson I.M., Berntorp E., Löfqvist T., Pettersson H. (1992). Twenty-five years’ experience of prophylactic treatment in severe haemophilia A and B. Intern Med.

[2] Gringeri A., Lundin B., von Mackensen S., Mantovani L., Mannucci P.M., ESPRIT Study Group (2011). A randomized clinical trial of prophylaxis in children with hemophilia A (the ESPRIT Study). J Thromb Haemost.

[3] Manco-Johnson M.J., Abshire T.C., Shapiro A.D., Riske B., Hacker M.R., Kilcoyne R. (2007). Prophylaxis versus episodic treatment to prevent joint disease in boys with severe hemophilia. N Engl J Med.

[4] Rayment R., Chalmers E., Forsyth K., Gooding R., Kelly A.M., Shapiro S. (2020). British Soc. for Haematology. Guidelines on the use of prophylactic factor replacement for children and adults with haemophilia A and B. Br J Haematol.

[5] Scott M.J., Xiang H., Hart D.P., Palmer B., Collins P.W., Stephensen D. (2019). Treatment regimens and outcomes in severe and moderate haemophilia A in the UK: the THUNDER study. Haemophilia.

[6] Skinner M.W., Négrier C., Paz-Priel I., Chebon S., Jiménez-Yuste V., Callaghan M.U. (2021). The effect of emicizumab prophylaxis on long-term, self-reported physical health in persons with haemophilia A without factor VIII inhibitors in the HAVEN 3 and HAVEN 4 studies. Haemophilia.

[7] Oldenburg J., Mahlangu J.N., Kim B., Schmitt C., Callaghan M.U., Young G. (2017). Emicizumab prophylaxis in hemophilia A with inhibitors. N Engl J Med.

[8] Kitazawa T., Igawa T., Sampei Z., Muto A., Kojima T., Soeda T. (2012). A bispecific antibody to factors IXa and X restores factor VIII hemostatic activity in a hemophilia A model. Nat Med.

[9] Mahlangu J., Oldenburg J., Paz-Priel I., Negrier C., Niggli M., Mancuso M.E. (2018). Emicizumab prophylaxis in patients who have hemophilia A without inhibitors. N Engl J Med.

[10] Young G., Liesner R., Chang T., Sidonio R., Oldenburg J., Jiménez-Yuste V. (2019). A multicenter, open-label phase 3 study of emicizumab prophylaxis in children with hemophilia A with inhibitors. Blood.

[11] Pipe S.W., Shima M., Lehle M., Shapiro A., Chebon S., Fukutake K. (2019). Efficacy, safety, and pharmacokinetics of emicizumab prophylaxis given every 4 weeks in people with haemophilia A (HAVEN 4): a multicentre, open-label, non-randomised phase 3 study. Lancet Haematol.

[12] Callaghan M.U., Negrier C., Paz-Priel I., Chang T., Chebon S., Lehle M. (2021). Long-term outcomes with emicizumab prophylaxis for hemophilia A with or without FVIII inhibitors from the HAVEN 1-4 studies. Blood.

[13] Lenting P.J., Denis C.V., Christophe O.D. (2017). Emicizumab, a bispecific antibody recognizing coagulation factors IX and X: how does it actually compare to factor VIII?. Blood.

[14] Makris M., Iorio A., Lenting P.J. (2019). Emicizumab and thrombosis: the story so far. J Thromb Haemost.

[15] Wall C., Xiang H., Palmer B., Chalmers E., Chowdary P., Collins P.W., UK Haemophilia Centre Doctors’ Organisation (UKHCDO) (2023). Emicizumab prophylaxis in haemophilia A with inhibitors: three years follow-up from the UK Haemophilia Centre Doctors' Organisation (UKHCDO). Haemophilia.

[16] Hay C.R.M., Xiang H., Scott M., Collins P.W., Liesner R., Dolan G. (2017). The haemtrack home therapy reporting system: design, implementation, strengths and weaknesses: a report from UK Haemophilia Centre Doctors Organisation. Haemophilia.

[17] Saxena G.K., Theocharopoulos I., Aziz N.T., Jones M., Gnanapavan S., Giovannoni G. (2020). GloBody technology: detecting anti-drug antibody against VH/VL domains. Sci Rep.

[18] Blanchette V.S., Key N.S., Ljung L.R., Manco-Johnson M.J., van den Berg H.M., Srivastava A. (2014). Subcommittee on Factor VIII, Factor IX and Rare Coagulation Disorders of the Scientific and Standardization Committee of the International Society on Thrombosis and Hemostasis. Definitions in hemophilia: communication from the SSC of the ISTH. J Thromb Haemost.

[19] Hart D.P., Alamelu J., Bhatnagar N., Biss T., Collins P.W., Hall G. (2021). Immune tolerance induction in severe haemophilia A: a UKHCDO inhibitor and paediatric working party consensus update. Haemophilia.

[20] Hilliard P., Funk S., Zourikian N., Bergstrom B.M., Bradley C.S., McLimont M. (2006). Hemophilia joint health score reliability study. Haemophilia.

[21] Kuijlaars I.A.R., Timmer M.A., de Kleijn P., Pisters M.F., Fischer K. (2017). Monitoring joint health in haemophilia: factors associated with deterioration. Haemophilia.

[22] Herdman M., Gudex C., Lloyd A., Janssen M., Kind P., Parkin D. (2011). Development and preliminary testing of the new five-level version of EQ-5D (EQ-5D-5L). Qual Life Res.

[23] Cleeland C.S. (2009).

[24] Palmer B., Xiang H., Hay C. UKHCDO annual report 2023 and bleeding disorder statistics for the financial year 20232/2024. https://www.ukhcdo.org/wp-content/uploads/2024/12/UKHCDO-Annual-Report-2024-2023-24-Data.pdf.

[25] Mahlangu J., Oldenburg J., Callaghan M.U., Shima M., Santagostino E., Moore M. (2018). Bleeding events and safety outcomes in persons with haemophilia A with inhibitors: a prospective, multi-centre, non-interventional study. Haemophilia.

[26] Batsuli G., Greene A., Meeks S.L., Sidonio R.F. (2021). Emicizumab in tolerized patients with hemophilia A with inhibitors: a single-institution pediatric cohort assessing inhibitor status. Res Pract Thromb Haemost.

[27] Dubé E., Merlen C., Bonnefoy A., Pilon J., Zourikian N., Gauthier J. (2023). Switching to emicizumab: a prospective surveillance study in haemophilia A subjects with inhibitors. Haemophilia.

[28] Liu G., Huang K., Li G., Zhen Y., Li Z., Chen Z. (2022). Real-world experience of emicizumab prophylaxis in young children with hemophilia A: retrospective data from China. Front Pediatr.

[29] Doshi B.S., Witmer C.M. (2021). Recurrence of a high-titre factor VIII inhibitor in a haemophilia A patient on emicizumab prophylaxis. Haemophilia.

[30] Capdevila L., Borgel D., Lasne D., Lacroix-Desmazes S., Desvages M., Delignat S. (2021). Reappearance of inhibitor in a tolerized patient with severe haemophilia A during FVIII-free emicizumab therapy. Haemophilia.

[31] Hay C.R., DiMichele D.M., International Immune Tolerance Study (2012). The principal results of the International Immune Tolerance Study: a randomized dose comparison. Blood.

[32] Mariani G., Kroner B., Immune Tolerance Study Group (ITSG) (2001). Immune tolerance in hemophilia with factor VIII inhibitors: predictors of success. Haematologica.

[33] Dimichele D. (2009). The North American Immune Tolerance Registry: contributions to the thirty-year experience with immune tolerance therapy. Haemophilia.

[34] Schmitt C., Emrich T., Chebon S., Fernandez E., Petry C., Yoneyama K. (2021). Low immunogenicity of emicizumab in persons with haemophilia A. Haemophilia.

[35] Callaghan M., Negrier C., Paz-Priel I., Chebon S., Lehle M., Mahlangu J. (2019). Emicizumab treatment is efficacious and well tolerated long term in persons with haemophilia A (PwHA) with or without FVIII inhibitors: pooled data from four HAVEN studies [abstract]. ISTH Academy.

